# A Frame Detection Method for Real-Time Hand Gesture Recognition Systems Using CW-Radar

**DOI:** 10.3390/s20082321

**Published:** 2020-04-18

**Authors:** Myoungseok Yu, Narae Kim, Yunho Jung, Seongjoo Lee

**Affiliations:** 1 Dept. Information and Communication Engineering, Sejong University, Gunja-dong, Gwangjin-gu, Seoul 05006, Korea; myoungseok@itsoc.sejong.ac.kr (M.Y.); narae@itsoc.sejong.ac.kr (N.K.); 2 School of Electronics and Information Engineering, Korea Aerospace University, Goyang, Gyeonggi-do 10540, Korea; yjung@kau.ac.kr

**Keywords:** hand gesture, micro-Doppler signatures, CW radar, convolutional neural network, real-time process, detection

## Abstract

In this paper, a method to detect frames was described that can be used as hand gesture data when configuring a real-time hand gesture recognition system using continuous wave (CW) radar. Detecting valid frames raises accuracy which recognizes gestures. Therefore, it is essential to detect valid frames in the real-time hand gesture recognition system using CW radar. The conventional research on hand gesture recognition systems has not been conducted on detecting valid frames. We took the R-wave on electrocardiogram (ECG) detection as the conventional method. The detection probability of the conventional method was 85.04%. It has a low accuracy to use the hand gesture recognition system. The proposal consists of 2-stages to improve accuracy. We measured the performance of the detection method of hand gestures provided by the detection probability and the recognition probability. By comparing the performance of each detection method, we proposed an optimal detection method. The proposal detects valid frames with an accuracy of 96.88%, 11.84% higher than the accuracy of the conventional method. Also, the recognition probability of the proposal method was 94.21%, which was 3.71% lower than the ideal method.

## 1. Introduction

HUMAN-computer interaction (HCI) recognizes the purpose of humans and operates a device. Hand gesture recognition is a type of HCI that gets the hand gestures of humans sorted and steers the device for each gesture. According to the advance of hand gesture recognition, the hand gesture recognition system can be applied to various devices, such as drones and robots. There is a representative advantage in hand gesture recognition, so a user can control a device remotely. In particular, it is a great merit when steering a drone, because the flying is separated between a user and a drone. In addition, hand gesture recognition has an advantage, because the movement of the hand is intuitive. The intuitive hand movement does not require a special explanation for the user.

The research of hand gesture recognition is being studied and developed [1,2,3]. Hand gesture recognition systems based mainly on camera sensors and accelerometer sensors are widely used. Camera sensors shoot the hand gesture and go through the process of recognizing the photographed data. Since the photographed data contains shape information, it can be effectively utilized for hand gestures with various image processing techniques [4,5]. However, there are disadvantages that arise from using the photographed data such as recognition environment and privacy violations. For example, vision sensors such as cameras may have difficulty in recognition in severe environments such as foggy or dark place. Also, camera sensors may cause privacy violations if they are hacked or personal privacy can be filmed. Recently, noncooperative space targets detection is being studied with vision sensors [6,7]. The target can be detected by finding the pose (position and attitude) of the noncooperative space target. However, the shape of the target must be circular or near-circular and the algorithm cannot be applied to real-time recognition systems due to the large amount of computation. Accelerometer sensors will be also utilized with wearable devices like gloves or bracelet for hand gesture recognition [8,9]. Accelerometer sensors have the advantage of being able to recognize minute movements, such as the gestures of a finger, but have an inconvenience of always wearing a sensor. Radar sensors are less sensitive to recognition environments and privacy violations and are being actively studied for hand gesture recognition [10,11,12,13,14,15]. In addition, the sensors do not need to be worn unlike accelerometer sensors. Therefore, this paper also adopts radar sensors for hand gesture recognition.

When using a radar sensor, frequency modulated continuous wave (FMCW) and continuous wave (CW) radar are used [10,11,12]. The reason for using the FMCW radar and the CW radar is to measure the micro-Doppler signatures of hand gestures [13,14,15]. The measured micro-Doppler signal can generate spectrogram via short term Fourier transform (STFT). The spectrogram expresses the frequency component of a hand gesture with time in the image. Therefore, the spectrogram can be used to use powerful machine learning for image recognition, such as the convolutional neural network (CNN). Previous research has only focused on hand gesture recognition and not on detecting a valid frame [16,17,18]. A valid frame is a frame that contains whole information of hand gestures, while an invalid frame contains partial information of hand gestures or does not have any information. Hand gestures can be correctly recognized only after detecting a valid frame. It is very important to detect a valid frame in order to process the real-time hand gesture system. We undertook this study about a valid frame detection to construct the real-time hand gesture system.

Signal detection has been studied in many fields, such as communications, voice activity detection (VAD) and constant false alarm rate (CFAR). However, it is impossible to use the above detection methods for a real-time hand gesture recognition system using CW radar. Communication detection methods are not suitable for the system using CW radar, because they require signals, such as pilots or preambles. Also, the CW radar detection method is not appropriate for using a VAD method because of the difference of the voice and the CW radar signal. The VAD method requires a sustained time but the CW radar signal appears in the form of an impulse signal in a shorter time than the voice signal.

Real-time hand gesture recognition using FMCW radar employs an exponentially weighted moving average (EWMA), which is one of the CFAR algorithms used to detect valid frames [19]. EWMA defines detection as the change in average energy increases. Also, EWMA is not suitable for detecting sensitive energy changes. FMCW radar calculates signals by beat frequency, so it does not reflect energy fluctuations significantly. Thus, the hand gesture recognition system using FMCW radar can get a high accuracy when EWMA is applied as a detection method. On the other hand, CW radar calculates hand gestures with energy change, so it is sensitive to energy fluctuations. Therefore, a hand gesture recognition system that uses CW radar cannot use EWMA. Since the detection method described above cannot be used, the detection method of an electrocardiogram (ECG) was selected as a conventional method. A CW radar signal is similar to an R-wave on an ECG. Both signals appear in the form of an impulse signal in a short time. R-wave detection in ECG uses a shifting window difference threshold (SWDT) [20]. Hence, SWDT was selected as a conventional method. In this paper, methods were provided to detect frames that can be used in real-time hand gesture recognition using CW radar. Also, the performances of the proposed and conventional method were compared. Section 2 explains roughly the hand gesture recognition system using CW radar. The conventional method is entered into the details, and the proposed methods are presented in Section 2. Section 4 accounts for the experiment environment and displays the experimental result. Section 5 summarizes the results and concludes this paper.

## 2. System Overview

**Figure 1:**
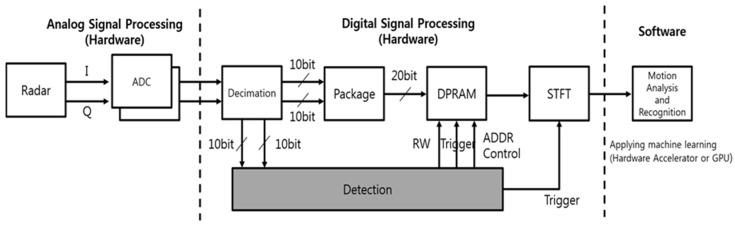
Diagram of Hand Gesture Recognition System Using CW radar Sensor.

Hand gesture recognition is a method of measuring hand gesture features from a sensor and analyzing and recognizing the measured data. Therefore, in the hand gesture recognition system, the sensor can be used in various ways. Among the various sensors, this paper describes a hand gesture recognition system using CW radar. Figure 1 shows a diagram of hand gesture recognition using CW radar. The hand gesture recognition system using CW radar is divided into three major parts: the analog signal processing part, the digital signal processing part and the software part.

The analog signal processing part measures the micro-Doppler signatures of the hand gesture from the CW radar and converts the analog signals to the digital signal. First, the analog signal processing part measures the hand gesture’s micro-Doppler signal through the CW radar. The micro-Doppler signal is measured on two channels as I/Q data. Then, the I/Q data are converted to a digital signal using an analog-to-digital converter (ADC) for digital signal processing.

The digital signal processing part processes digital signals converted through the ADC. First, the digital signal which is transmitted by the analog signal processing part is preprocessed. Pre-Processing includes processing that proceeds to easily signal process the raw data coming in from the ADC. The pre-processing module consists of Decimation, Re-formation. The reason for proceeding with decimation is that using a sampling rate that is too high can increase the complexity and have processing times that are too high relative to the accuracy of the spectrogram. The decimated signal proceeds through the re-formation process. Re-formation is the process of converting the signal coming from the ADC to make it easier to perform bit operations. Pre-processed signals are connected on both sides by package and detection. The package is a module that combines I/Q data into one. The reason why I/Q data from the package is put together is to make it easy to save and access data measured at the same time of dual-ported random-access memory (DPRAM). The data generated from the package is transferred and stored to the space of DPRAM appointed by each ADDRESS. When a valid frame is detected, the detection module analyzes the received decimation signal to determine if a valid frame has been detected. Then the detection module informs the DPRAM and the STFT modules that the available frame has been detected via the trigger signal. The detection module also notifies the DPRAM of the ADDRESS where there is a valid frame of data stored in the DPRAM via the ADDRESS control signal. Finally, the detection module indicates whether to write that it is to read data from the DPRAM using the R/W signal. The DPRAM module saves and outputs decimation data based on the signal from the detection. The STFT module makes from a valid frame to the spectrogram. When the trigger signal comes in, the STFT module receives a valid frame from the DPRAM; this frame is output to spectrogram by STFT. The software part analyzes and recognizes motion using the spectrogram transmitted by the digital signal processing part. The software part performs machine learning using a hardware accelerator or a graphics processing unit (GPU) on the spectrogram. It makes possible to recognize any motion according to the spectrogram using a machine learning model generated through machine learning.

In this paper, a CNN model was employed as the software part. The CNN model was trained by the spectrogram of hand gestures. Then, hand gestures were inferred using the CNN model. The inference was taken within 0.1 s using a computer. It is enough time for the real-time process.

## 3. Detection Methods

**Figure 2:**
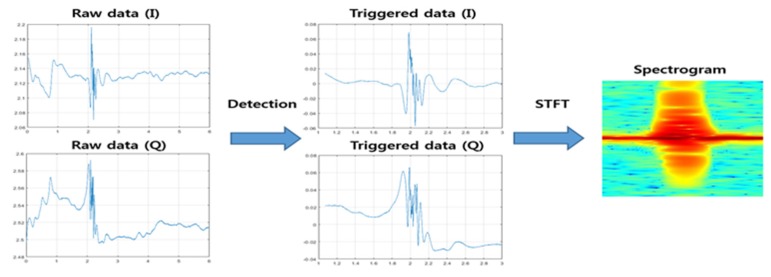
Example of detection.

In this section, the conventional method [20] and the proposed method are presented. The conventional method calculates a trigger value from the raw data. Then, a trigger value is compared with the threshold. The proposed method consists of a 2-stage detection method. The first stage of the proposed method roughly detects the valid data. After that, the second stage proceeds with detection where it has the largest energy near the detection position of the first stage. Figure 2 shows a spectrogram created on the valid frame data detected in raw data.

The conventional method divides the raw data into frames and calculates the trigger value. First, to calculate the trigger value, which is shown in Equation (1), *D* is calculated, which means the difference between adjacent samples in the frame. In Equation (1), *F* means the value of the sample present in the frame and abs means the absolute value in Equation (1). The absolute value of the complex number is the square root of the sum of the values where the real value and the imaginary value are, respectively, squared. However, the absolute magnitude of a complex number can be obtained without using the square root as in Equation (2). Therefore, abs use Equation (2) in the conventional method to reduce the computational complexity.

(1) D[k]=abs(F[k]−F[k+1])

(2) $$abs\left(R\right)=I^{2}+Q^{2}$$

*D* is calculated for all values in a frame. Therefore, the value in the range corresponding to k = (1, 2,… sizeof(F)) is calculated. Also, all the calculated D values are summed. Equation (3) shows a value obtained by adding up all the *D* values of the frame. The value of Equation (3) is defined as the trigger value.

(3) $$T\;=\overset{n}{\underset{k=1}{\sum }}D\left[k\right]$$

After calculating the trigger value, the trigger value compares the magnitude with a threshold. If the trigger value is smaller than the threshold, the next frame recalculates the trigger value. Conversely, when the trigger value is greater than the threshold, it is defined that a valid frame is detected. Since the conventional method has a threshold set to a constant and the trigger value exists as an absolute numerical value, there is a disadvantage that the detection probability may change depending on the magnitude of the hand movement.

The proposed methods consist of two stages to compensate for and overcome the disadvantages of the conventional method, thereby increasing the detection probability. Stage 1 searches for an approximate valid frame location. The frames existing around the frame position detected in Stage 1 are defined as the window. Stage 2 proceeds in a way that finds the maximum trigger value in the window.

**Figure 3:**
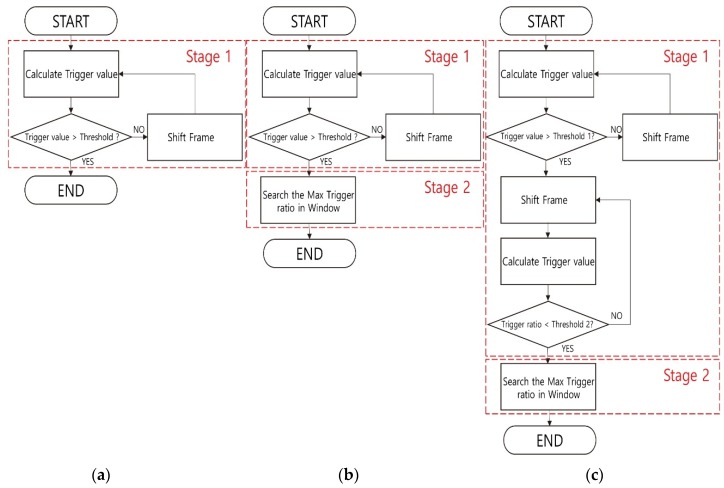
Flowchart of proposed methods. (**a**) conventional method; (**b**) proposed method 1; (**c**) proposed method 2.

The proposed method 1 uses the conventional method in Stage 1. In the proposed method 1a window is defined as a frame located behind in time in the frame detected in Stage 1. The reason for defining the window by the above method is that it is not necessary to recalculate the trigger value, which is calculated previously. Stage 2 calculates the trigger value of the frame present in the window, and then a frame that has the largest trigger value in the window is defined as a valid frame. As shown in Figure 3, the conventional method consists of single stage and determines the valid frame by using the frame with a trigger value exceeding the threshold. However, the proposed method 1 uses the trigger values of all frames within the window in Stage 2 and finds out the valid frame, which results in getting high accuracy than the conventional method. The proposed method 2 detects in Stage 1 using the rate of change of the trigger value. The change ratio of the trigger value is defined as the trigger ratio; the trigger ratio is calculated as shown in Equation (4). The trigger ratio is calculated using the trigger value calculated in a present frame and the trigger value of a frame existing after delay. T[n] means the present trigger value and T [n+delay] means the delayed trigger value in Equation (4). The trigger ratio does not grasp the magnitude of the trigger value, but it grasps the change ratio of the trigger value. Therefore, the method using the trigger ratio has an advantage, because the detection probability doesn’t change according to the magnitude of the hand movement. In this paper, the delay was 50 milliseconds, a half of the size of window 1.

(4) $$R=\frac{T\left[n+delay\right]}{T\left[n\right]}$$

**Figure 4:**
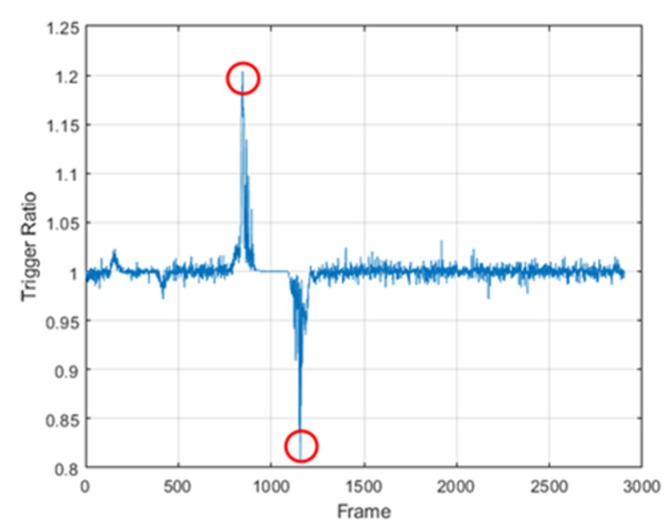
Example of the trigger ratio.

The trigger ratio calculated via (4) is displayed in Figure 4. The trigger ratio can find the starting point of the hand gesture and the endpoint of the hand gesture. Looking at Figure 4, the trigger ratio keeps a value of around 1 but increases to more than 1 significantly later. The reason the trigger ratio is kept around 1 is that a delayed trigger value and the present trigger value are almost equal. Before the hand gesture is started, the delayed trigger is kept similar to the present trigger value. The reason for an increase in the trigger ratio is that the delayed trigger value is bigger than the present trigger value. When the hand gesture is started, the delayed trigger increases. The high peak of the trigger ratio means the hand gesture is begun. The red circle at the upper left side in Figure 4 signifies a start point. After this, the trigger ratio reduces to around 1, and it maintains its value. While the hand gesture is sustained, the present trigger value increases as much as the delayed trigger value. Then, the trigger ratio decreases to less than 1, because the delayed trigger value is smaller than the present trigger value. When the hand gesture ends, the delayed trigger decreases. The red circle at the lower right side in Figure 4 signifies the low peak of the trigger ratio, which means that the hand gesture has ended. The threshold of the proposed method 2 has a threshold of the start point and a threshold of the endpoint. The threshold of the point where the hand gesture starts is defined as threshold 1, which has a value of more than 1. Also, the threshold of the point where the hand gesture ends is defined as threshold 2 which has a value of less than 1. The proposed method 2 detects the start point and the endpoint using both thresholds in first stage. When the trigger ratio exceeds threshold 1, that point is defined as the start point. After that, when the trigger ratio is lower than threshold 2, that point is defined as the endpoint. The middle point of the start points, and the endpoint is defined as the temporary valid frame. The window of proposed method 2 is defined as a frame before and a frame after the temporary valid frame. The reason for defining the window by the above method is that it is not possible to know whether the frame that has the maximum trigger value exists before or after the temporary valid frame. Stage 2 of proposed method 2 detects a frame that has a maximum trigger value in the window like proposed method 1. The frame having the largest trigger value in the second stage is defined as a valid frame. The method using the trigger ratio cannot detect the frame with the largest trigger value accurately but considers the relative change of data, so finding the approximate position of the frame with the largest trigger value is powerful. In this paper, we experimented with assuming real-time processing. Real-time processing requires a short processing time. Therefore, real-time processing is impossible if the window size is too long. For the above reasons, the window size was fixed within 0.1 s and 0.2 s in the second stage of the proposed methods. A window with a size of 0.1 s is defined as window 1; a window with a size of 0.2 s is defined as window 2.

## 4. Experiment

This section describes experiments to confirm the performance of the proposed methods and analyzes the results. First, the experimenter’s hand gestures were measured as a micro-Doppler signal via the CW radar. Next, the measured data were created in a spectrogram and the CNN model was trained using the spectrogram. The detection range was defined using the CNN model and the raw data. The detection probability was the result of calculating the probability that the valid frame of each method was included within the detection range. Finally, spectrograms made from the valid frame of each method calculate the recognition probability through the inference process of the CNN model. In the experiment, a K-MC1 radar, ST200 hardware and RFbeam signal explorer software were used. The operating frequency of the K-MC1 was 24 GHz (K-Band), and it uses 180 MHz sweep FM (frequency modulation) input. The ST200 has a 16-bit data acquisition at the speed of 250 ksps (sample per second) sampling rate. We employed 6 hand gestures.

**Figure 5:**
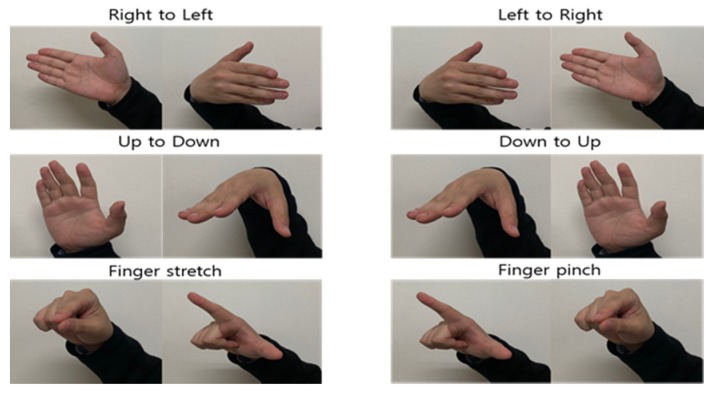
Types of hand gestures.

Figure 5 shows the hand gestures. The six hand gestures consist of right-to-left, left-to-right, up-to-down, down-to-up, finger stretch and finger pinch. The distance to the hand was set to about 30 cm in the radar module. Also, we measured the micro-Doppler signal of the hand gesture in front of the radar module. The gestures of right-to-left and left-to-right consisted of 45 degrees of movement from the radar line of sight. The gestures of Up to down and Down to up had 45 degrees of movement from the horizontal position of the radar. The maximum angle of outstretched fingers was 90 degrees for finger gestures. Four people took part in the experiments. Before measuring the raw data, each hand gesture was ready to the initial pose as shown in the left part of Figure 5. Each hand gesture was performed with undetermined time delay after the start of data gathering system. One person did repeatedly an action 100 times for each gesture, which resulted in the collection of 400 data for each gesture. The raw data were measured for 6 s, in which the hand gesture frames were included for 1.8 s with unknown time position. The starting point (valid frame) of hand gesture should be correctly determined by the detection algorithm before the start of recognition process.

**Figure 6:**
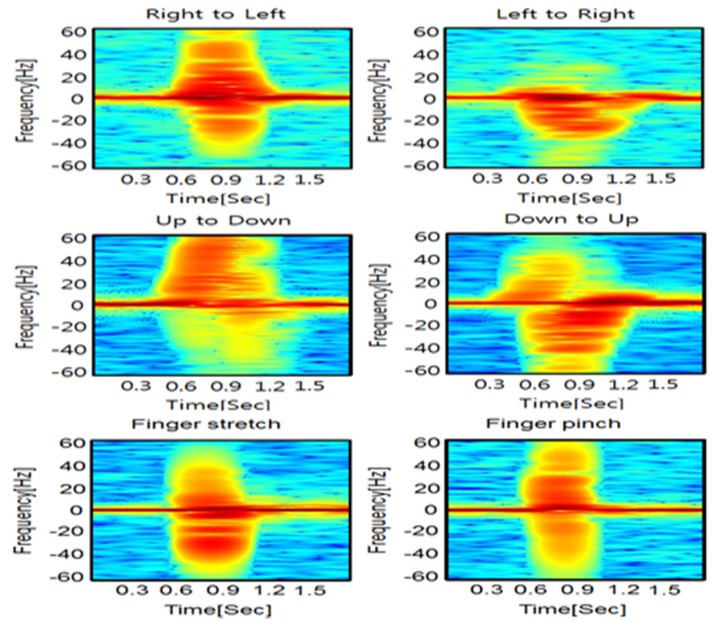
Spectrograms of each hand gesture.

Figure 6 depicts the spectrogram of each of the hand gestures. The conditions of STFT in this paper were as follows. The size of the FFT configured in STFT was 512 samples, the number of non-overlapping samples was 5 samples; color mapping uses jet color mapping [18]. The spectrogram of Figure 6 used the ideal method. The ideal method detects a frame that has a maximum trigger value within 6 s of the measured raw data. In the ideal method, all data samples of Micro-doppler signal were stored and evaluated from the start of experiment to the end of it before finding the valid frame. The ideal method can compare the overall Micro-doppler signal data and show the best detection performance, but cannot be performed in real-time, because it has to be waiting for the end of entire data gathering process. Also, the detection range was defined based on a frame detected using the ideal method.

**Figure 7:**
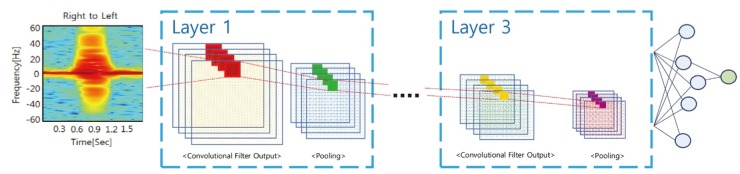
Structure in convolutional neural network (CNN) with three layers.

As shown at Figure 7, the convolutional neural network (CNN) model was constructed using a spectrogram created using the ideal method. We utilized the data for training with 90% of the obtained micro-Doppler signals. And the rest of the measured data were used for both the inference and the accuracy estimation. The CNN consisted of three convolutional layers, each of which contained the combination of convolution filters, an activation function and a pooling. Through experiment optimization, 16 convolution filters and an average-pooling scheme were used. An activation function was a sigmoid function.

**Figure 8:**
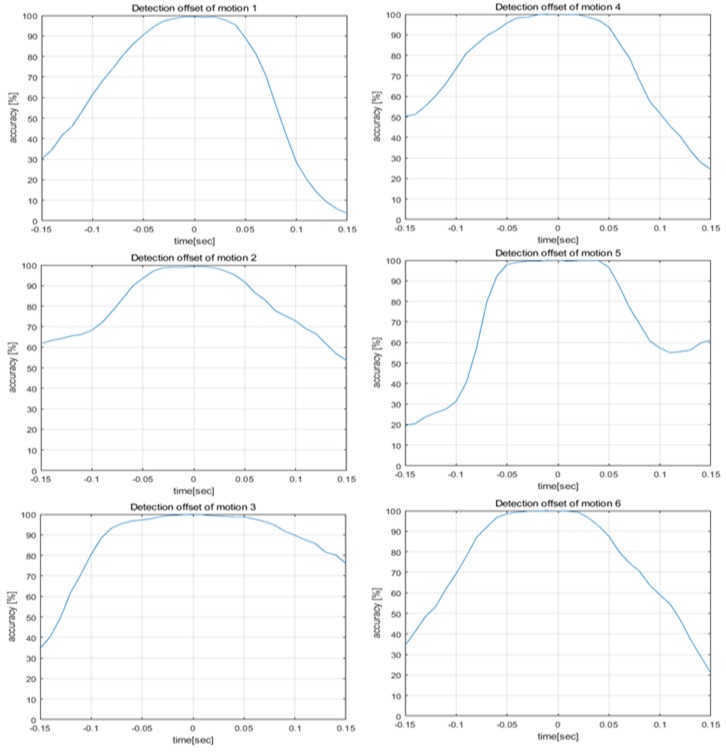
The performance drops caused by detection offset in motion 1 to 6.

Figure 8 displays the performance drop according to the detection offset. To define the detection range, we used the performance drop according to the detection offset. The frame detected by the ideal method was set to 0 in the *x*-axis in Figure 8. Detection offset means the error frame. Error frames were made with an error in 0.01 s units based on the ideal method. Then, error frames were inferred using the CNN model and calculated the recognition probability. Recognition probability was defined as the accuracy when the inference of pre-trained deep learning model was correct from the data detected from a spectrogram. Commercial recognition systems require more than 95% accuracy, so we established a detection range where the performance drop was up to 5%.

**Figure 9:**
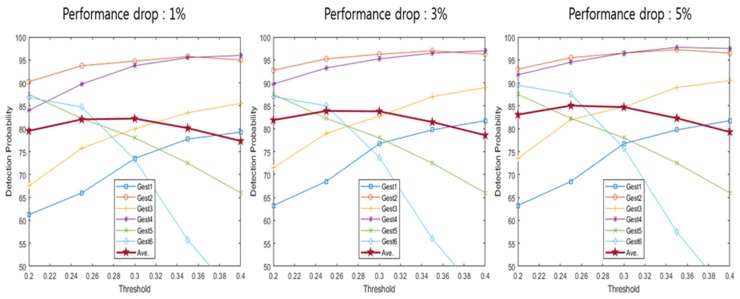
Detection probability of conventional method [20].

**Figure 10:**
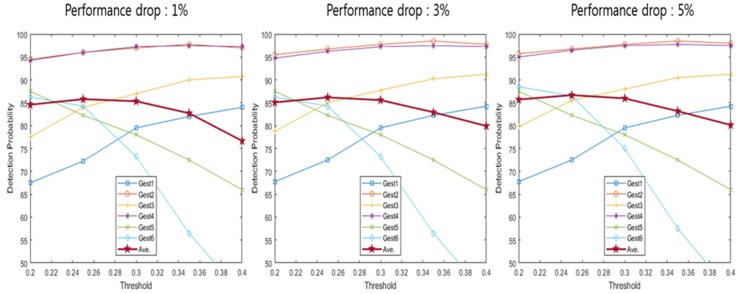
Detection probability of proposed method 1 (window 1).

**Figure 11:**
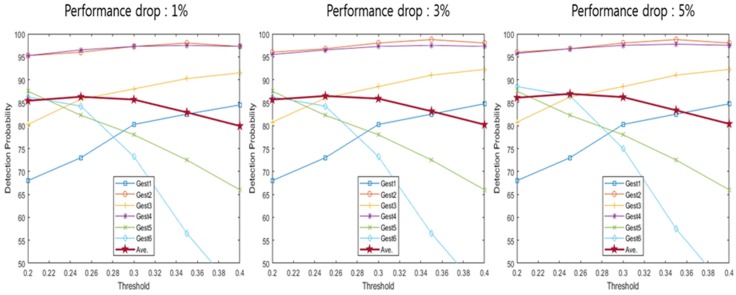
Detection probability of proposed method 1 (window 2).

**Figure 12:**
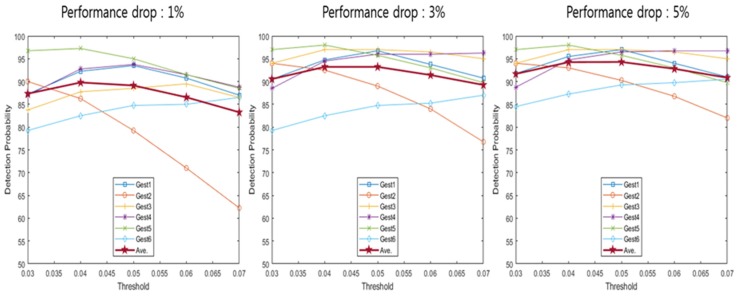
Detection probability of proposed method 2 (window 1).

**Figure 13:**
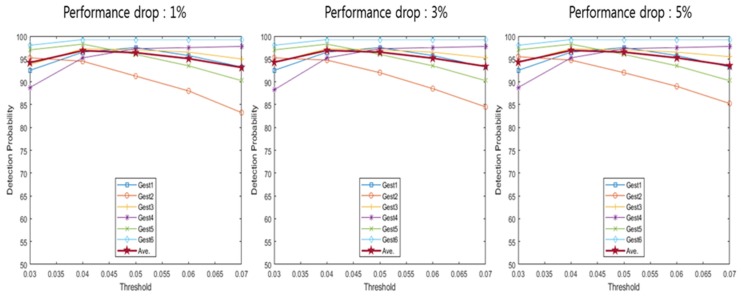
Detection probability of proposed method 2 (window 2).

Table 1 indicates a detection range based on the performance drop according to the detection offset. The detection range was calculated for each detection offset where the performance drops were 1%, 3% and 5%. Detection probability uses the detection range described in Table 1 to calculate the probability that the spectrogram configured using each method enters the detection range. Figure 9, Figure 10, Figure 11, Figure 12 and Figure 13 depict the detection probability of each method. Each graph was created according to the detection range for each offset. A window does not exist in the conventional method, but the proposed method consists of a 2-stage and a window exists. The size of window 1 was 0.1 s; the size of window 2 was 0.2 s. The window of the proposed method 1 consists of a frame after the frame detected in the first stage and proposed method 2 consists of a frame before and a frame after the frame detected in the first stage. Looking at Figure 9′s conventional method detection probability, it can be seen that the detection probability changes significantly based on each threshold. The gestures 1–4 were large movements that use the whole hand, so it was possible to see a high detection probability when the threshold was high, but a low detection probability when the threshold was low. However, since the gestures 5–6 were small movements using only two fingers, it can be seen that the detection probability was low when the threshold was large, and detection probability was high when the threshold was small. Large gestures cause the trigger value to fluctuate more than small gestures. Therefore, there was a probability that early detection occurs when the threshold was small for large gestures. Early detection means an error detecting a frame exists before a valid frame. Conversely, small gestures were indicated by small changes in the trigger value. Thus, small gestures have a probability that a miss will occur if the threshold was large. A miss means passing without detecting a valid frame. As each performance drop increases, the detection range expands more. Therefore, when the performance drop was high, it can be seen that the detection probability becomes the same as or higher than when the performance drop was low. The conventional method has the advantage of being the simplest over the other methods However, the conventional method has the disadvantage of a low detection probability. Looking at Figure 9, Figure 10 and Figure 11 and Table 2, Table 3 and Table 4, the proposed method 1 shows a higher detection probability than the conventional method, because the first stage was added in the conventional method. Also, when the window size was 0.2, the detection probability was higher than when the window size was 0.1. The reason why the detection probability was high when the window size was large was it has found the maximum value of the trigger value in a wider range. However, if the window size was large, it may need more time with the real-time process, so the difference in the size of the window size has advantages and disadvantages. The detection and recognition probabilities of the proposed method 1 increase up to 86–92% and up to 86−67%, respectively. The proposed method 1 shows a higher performance than the conventional method. However, since Stage 1 of the proposed method 1 uses the conventional method, the detection probability and the recognition probability change greatly according to the magnitude of the threshold. As described in the previous conventional method, a large gesture indicates a higher detection probability if the threshold was large; a small gesture indicates a higher detection probability if the threshold was small in the proposed method 1. Therefore, the proposed method 1 has problems such as the conventional method. After all, the proposed method 1 shows a higher performance than the conventional method, but it shows a performance that was not sufficient to configure the recognition system.

The proposed method 2 has no significant difference in the detection probability of a specific hand gesture based on the threshold. This was because the proposed method 2 does not use the trigger value but uses the trigger ratio. The trigger ratio signifies the relative change of the trigger value, so there was no difference between a large hand gesture and a small hand gesture. The proposed method 2 shows a higher detection probability than the conventional method and the proposed method 1, and was not limited to a specific hand gesture. Also, it shows a detection probability of about 90% for all hand gestures. In summary, from the results of Figure 9, Figure 10, Figure 11, Figure 12 and Figure 13, Table 2, Table 3, Table 4, Table 5 and Table 6 depict the averaged detection probabilities of all hand gestures for the methods evaluated with varying threshold values. As shown in Table 2, Table 3, Table 4, Table 5 and Table 6, we found that the optimum threshold values having the maximum detection probabilities were changed depending on not only the offset values but the detection methods. For example, as shown in Table 7, in the conventional method [20], the optimum threshold value was 0.3 when the offset value was 1%. At 3% offset, the optimal value of threshold was changed to 0.25. Hence, the optimal detection can find the positions having the maximum recognition probability.

Table 7 indicates the optimum threshold with the highest detection probability. The detection probability and the recognition probability of each method were compared using the optimum threshold.

Table 8 displays the detection probability of each method; Table 9 displays the recognition probability of each method. To measure the performance of the proposed method, Table 8 shows how much the detection probability was improved over conventional methods; Table 9 presents the recognition performance of proposed methods. The percentage in parentheses shows the performance drop compared with the method adopting the ideal (perfect) detection. The existing detection methods provided robustness in terms of false alarm. However, they had a weakness to miss the correct frames. Unlike the existing algorithms, the proposed method could have a strength with respect to the miss issue without increasing false alarm probabilities. In order to verify the false alarm issue, we measured the micro-Doppler signal for 6 seconds including hand gesture and inactivity. During the idle time, we evaluated whether or not the proposed method declared false alarms. In addition, the two-stage processing in the proposed scheme could have more reliable performance in terms of position accuracy of valid frames compared with the existing algorithms. The two proposed methods have a higher performance than the convention method. In particular, the proposed method 2 improves the detection probability by about 10% compared to the conventional method and decreases the recognition probability by 5% compared to the ideal method.

**Figure 14:**
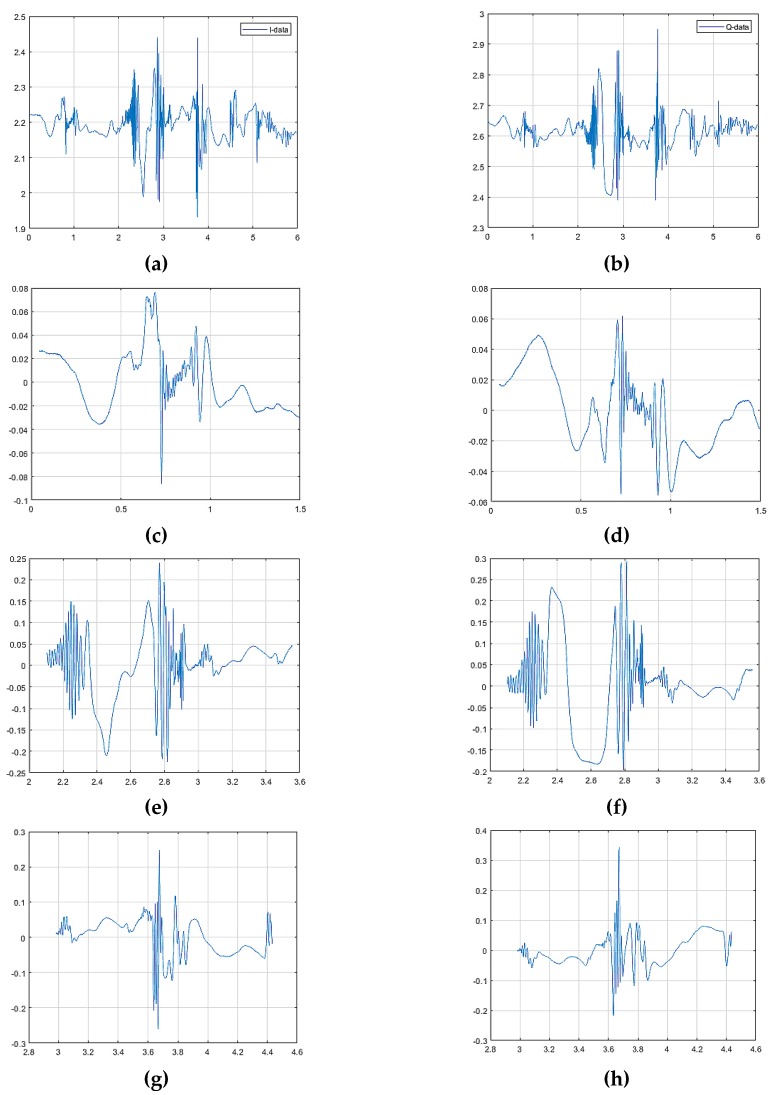
Detection probability of multiple hand gestures: (**a**) I-data of raw data; **(b**) Q-data of raw data; (**c**) I-data of the first hand gesture; (**d**) Q-data of the first hand gesture; (**e**) I-data of the second hand gesture; (**f**) Q-data of the second hand gesture; (**g**) I-data of the third hand gesture; (**h**) I-data of the third hand gesture.

**Figure 15:**
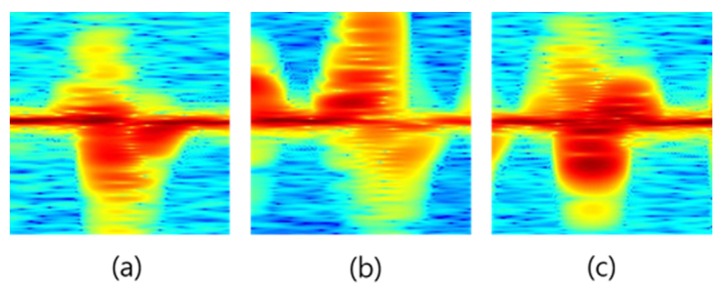
Spectrograms of multiple hand gestures: (**a**) spectrogram of the first hand gesture (right-to-left); (**b**) spectrogram of the second hand gesture (up-to-down); (**c**) spectrogram of the third hand gesture (finger stretch).

In order to verify the suitability for real-time processing, experiments were also conducted on the detection of raw data with multiple hand gestures and Table 10 shows the computational time of each methods. Since the proposed method 2 consists of two stages, it was about 0.06 s slower than the conventional method. However, the proposed method 2 can improve both a detection probability (96.88%) and a recognition probability (94.21%) significantly compared to the conventional one even satisfying real-time processing requirements. The computational time was measured on an Intel Core i7-8700 CPU and 16 G RAM. The experiments measured micro-Doppler signals of three hand gestures for 10 s. The measurements about continuous three hand gestures were performed for 100 times, and the proposed method 2 was evaluated in the experiments. Figure 14 shows an example of valid frame detection results for three hand gestures from the micro-Doppler signals containing three hand gestures. Figure 14a depicts the raw data of the micro-Doppler signals with continuous three hand gestures; we can see three detected gestures from Figure 14b–d. Figure 15 shows the spectrograms of three gestures, which were obtained from the detected frames. As shown in Figure 15, we can find that the spectrograms of all gestures were clearly generated. The detection probability was 93.33%.

## 5. Conclusions

In this paper, we proposed a method to detect frames that can be used to construct a real-time hand gesture recognition system using CW radar. To measure the performance of the proposed method, experiments were performed to measure hand gesture with CW radar. Hand gesture performed four hundred measurements in six divisions. The measured hand gesture was created in spectrogram; the CNN model was constructed using that spectrogram. The recognition probability and the detection probability were defined using the constructed CNN model. The conventional method has a detection probability of up to 85.04% and a recognition probability of up to 85.29%. Also, the proposed method 1 has a detection probability of up to 86.92% and a recognition probability of up to 86.67%. Therefore, the conventional method and the proposed method 1 show a performance that was not sufficient to construct a real-time hand recognition system using CW radar. The proposed method 2 has a detection probability of up to 96.88% and a recognition probability of up to 94.21%. In conclusion, based on these performance measurements, we believe that the proposed method 2 could be used to construct a real-time hand gesture recognition system using CW radar. Future research will create a detection method that exhibits higher detection probability to construct a real-time hand gesture recognition system using CW radar. In the direction of other research, we plan to take a wide detection range and construct a CNN model that can improve the recognition probability even using a simple detection method.

## Figures and Tables

**Table 1:** Detection range of each hand gesture.

Hand Gesture	Detection Range [sec]		
Perf. Drop	1% Drop	3% Drop	5% Drop
1	−0.02–0.03	−0.04–0.04	−0.04–0.05
2	−0.02–0.02	−0.04–0.04	−0.05–0.05
3	−0.03–0.03	−0.06–0.07	−0.08–0.08
4	−0.03–0.03	−0.05–0.04	−0.06–0.05
5	−0.04–0.05	−0.06–0.05	−0.06–0.06
6	−0.05–0.03	−0.06–0.03	−0.07–0.04

**Table 2:** Averaged detection probabilities of conventional methods [20].

Offset	Threshold				
	0.4	0.35	0.3	0.25	0.2
1%	77.33%	80.13%	82.21%	82.04%	79.54%
2%	78.58%	81.46%	83.79%	83.88%	81.88%
3%	79.29%	82.29%	84.71%	85.04%	83.08%

**Table 3:** Averaged detection probabilities of proposal 1 (window 1).

Offset	Threshold				
	0.4	0.35	0.3	0.25	0.2
1%	79.67%	82.71%	85.33%	85.79%	84.58%
2%	79.92%	82.92%	85.58%	86.17%	85.08%
3%	80.13%	83.17%	85.96%	86.67%	85.71%

**Table 4:** Averaged detection probabilities of proposal 1 (window 2).

Offset	Threshold				
	0.4	0.35	0.3	0.25	0.2
1%	79.92%	82.88%	85.67%	86.29%	85.42%
2%	80.21%	83.13%	85.88%	86.46%	85.67%
3%	80.38%	83.33%	86.21%	86.92%	86.08%

**Table 5:** Averaged detection probabilities of proposal 2 (window 1).

Offset	Threshold				
	±0.07	±0.06	±0.05	±0.04	±0.03
1%	83.25%	86.54%	89.13%	89.79%	87.33%
2%	89.25%	91.42%	93.21%	93.21%	90.54%
3%	90.83%	92.79%	94.29%	94.25%	91.67%

**Table 6:** Averaged detection probabilities of proposal 2 (window 2).

Offset	Threshold				
	±0.07	±0.06	±0.05	±0.04	±0.03
1%	93.13%	95.08%	96.38%	96.83%	94.21%
2%	93.38%	95.17%	96.50%	96.83%	94.29%
3%	93.54%	95.25%	96.50%	96.83%	94.33%

**Table 7:** Optimum thresholds of methods.

Offset	Conventional	Proposal 1		Proposal 2	
	-	Window1	Window2	Window1	Window2
1%	0.3	0.25	0.25	1 ± 0.04	1 ± 0.04
2%	0.25	0.25	0.25	1 ± 0.04	1 ± 0.04
3%	0.25	0.25	0.25	1 ± 0.05	1 ± 0.04

**Table 8:** Detection probability of methods.

**Offset**	**Proposal 1**	
	**Window 1**	**Window2**
1%	85.79% (3.58%↑)	86.29% (4.08%↑)
2%	86.17% (2.29%↑)	86.46% (2.58%↑)
3%	86.67% (1.63%↑)	86.92% (1.88%↑)
**Offset**	**Proposal 2**	
	**Window 1**	**Window2**
1%	89.79% (7.58%↑)	96.83% (14.62%↑)
2%	93.21% (9.33%↑)	96.88% (13.00%↑)
3%	94.29% (9.25%↑)	96.88% (11.84%↑)

**Table 9:** Recognition probability of methods.

**Gesture**	**Proposal 1**	
	**Window 1**	**Window2**
1	72.50% (26.00%↓)	72.50% (26.00%↓)
2	87.50% (8.00%↓)	87.50% (8.00%↓)
3	85.00% (13.00%↓)	84.75% (13.25%↓)
4	94.50% (3.00%↓)	94.75% (2.75%↓)
5	88.25% (11.00%↓)	90.00% (9.25%↓)
6	90.50% (8.25%↓)	90.50% (8.25%↓)
Ave.	86.38% (11.54%↓)	86.67% (11.25%↓)
**Gesture**	**Proposal 2**	
	**Window 1**	**Window2**
1	96.25% (2.25%↓)	96.00% (2.50%↓)
2	85.75% (9.75%↓)	87.00% (8.50%↓)
3	93.75% (4.25%↓)	94.50% (3.50%↓)
4	94.50% (3.00%↓)	93.25% (4.25%↓)
5	96.25% (3.00%↓)	97.75% (1.50%↓)
6	96.00% (2.75%↓)	96.75% (2.00%↓)
Ave.	93.75% (4.17%↓)	94.21% (3.71%↓)

**Table 10:** Computational time of methods.

Conventional	Proposal 1		Proposal 2	
-	Window1	Window2	Window1	Window2
0.108715	0.109328	0.109716	0.163992	0.164874
